# Comprehensive Transcriptome Analysis Uncovers Hub Long Non-coding RNAs Regulating Potassium Use Efficiency in *Nicotiana tabacum*

**DOI:** 10.3389/fpls.2022.777308

**Published:** 2022-03-31

**Authors:** Xi Chen, Lin Meng, Bing He, Weicong Qi, Letian Jia, Na Xu, Fengqin Hu, Yuanda Lv, Wenjing Song

**Affiliations:** ^1^Key Laboratory of Tobacco Biology and Processing, Ministry of Agriculture, Tobacco Research Institute, Chinese Academy of Agricultural Sciences (CAAS), Qingdao, China; ^2^Excellence and Innovation Center, Jiangsu Academy of Agricultural Sciences, Nanjing, China

**Keywords:** potassium, LncRNA, transcriptional regulation, co-expression network, *Nicotiana tabacum* L.

## Abstract

Potassium (K) is the essential element for plant growth. It is one of the critical factors that determine crop yield, quality, and especially leaf development in tobacco. However, the molecular mechanism of potassium use efficiency (KUE), especially non-coding RNA, is still unknown. In this study, tobacco seedlings were employed, and their hydro-cultivation with K treatments of low and sufficient concentrations was engaged. Physiological analysis showed that low potassium treatment could promote malondialdehyde (MDA) accumulation and antioxidant enzyme activities such as peroxidase (POD), ascorbate-peroxidase (APX). After transcriptomic analysis, a total of 10,585 LncRNA transcripts were identified, and 242 of them were significantly differently expressed under potassium starvation. Furthermore, co-expression networks were constructed and generated 78 potential regulation modules in which coding gene and LncRNAs are involved and functional jointly. By further module-trait analysis and module membership (MM) ranking, nine modules, including 616 coding RNAs and 146 LncRNAs, showed a high correlation with K treatments, and 20 hub K-responsive LncRNAs were finally predicted. Following gene ontology (GO) analysis, the results showed potassium starvation inducing the pathway of antioxidative stress which is consistent with the physiology result mentioned above. Simultaneously, a part of detected LncRNAs, such as *MSTRG.6626.1*, *MSTRG.11330.1*, and *MSTRG.16041.1*, were co-relating with a bench of MYB, C3H, and NFYC transcript factors in response to the stress. Overall, this research provided a set of LncRNAs that respond to K concentration from starvation and sufficient supply. Simultaneously, the regulation network and potential co-functioning genes were listed as well. This massive dataset would serve as an outstanding clue for further study in tobacco and other plant species for nutrient physiology and molecular regulation mechanism.

## Introduction

Potassium, nitrogen, and phosphorus are the principal and most essential elements for plant nutrients (Clarkson and Hanson, 1980). Potassium ion (K^+^) plays several crucial roles in the whole life cycle of plant development (Wang and Wu, 2013). The available potassium element is limited around the world. The potassium mines detected worldwide are located only in a few countries, including Canada, Russia, Brazil, and China (Prakash and Verma, 2016). Therefore, improving crops in their K^+^ deficiency tolerance or utility efficiency is entirely meaningful in breeding research. K^+^ is involved in plant photosynthesis, enzyme activity, osmoregulation, electrical neutralization, pH and ion homeostasis, anion-cation balance, membrane electrical potential, protein and starch synthesis, sugar and nutrient transport, and stomatal movement (Clarkson and Hanson, 1980; Ache et al., 2000). Potassium deficiency in plants shows leaf veining, brown scorching, and leaf tips curling (Hafsi et al., 2014). The plant development will be delayed in the roots and shoots by potassium deficiency (Hafsi et al., 2014). In crop, potassium deficiency will lead to yield reduction, decreased tolerance to biotic and abiotic stress, which will cause further loss. K^+^ transporters have been illustrated by previous studies (Wang et al., 2021). Three families were reported, of which Shaker, TPK, and Kir are included (Very and Sentenac, 2003; Gambale and Uozumi, 2006; Lebaudy et al., 2007). The K^+^ in the plant cell is stored in the vacuole, and the concentration could be as high as 10 to 200 mM (Leigh and Wyn Jones, 1984; Walker et al., 1996). On the other hand, the K^+^ concentration in soil is as low as 0.1 to 1 mM (Schroeder et al., 1994; Maathuis, 2009). The plant absorbs K^+^ from the soil with the root. Hence, K^+^ is the most critical and principal part of fertilizer.

In plant cells, K^+^ is stored mainly by the vacuole. The K^+^ concentration in the cytoplasm keeps homeostasis because vacuolar K^+^ will be released when the deficiency in the cytoplasm occurs. CBL2/3 and CIPK9 mediated the exportation of K^+^ from the vacuole, but the channel has not yet been identified (Liu et al., 2012). Pyruvate kinase might be one of the sensors that control the efflux of vacuolar K^+^. Once K^+^ is activated, long-term condition of low K^+^ concentration could suppress its expression, further leading to decreased pyruvate content (Ramírez-Silva et al., 2001), further influencing relevant glycolysis pathways transducing K^+^ deficiency signal downward. Hence, although plants have developed a functional sink of K^+^ maintenance in evolution, plants would also suffer from low K^+^ concentration in soil. Long-term K^+^ deficiency would negatively affect plant growth and crop yield (Wang et al., 2021). Plants have a series of mechanisms to sense the environmental K^+^ sufficiency or deficiency. Typically, plant root is the organ in contact directly with soil. The potential of the root epidermis cytoplasm is the primary force driving K^+^ absorption. It is pretty sensitive to the K^+^ concentration decrease, which can cause membrane hyperpolarization. Membrane hyperpolarization could promote the activity of voltage-gated K^+^ channels, for example, Shaker AKT1-like (Czempinski et al., 2002). Membrane hyperpolarization could also activate Ca^2+^ permeable channels on the root epidermis. The Ca^2+^ influx could also be a signal in plant cells responding to K^+^ deficiency.

On the other hand, K^+^ deficiency might induce ROS generation. ROS and Ca^2+^ probably work together in the potassium starvation signal transduction (Maathuis and Sanders, 1993; Shin and Schachtman, 2004). Previous studies showed potassium starvation-induced oxidative stress in *Arabidopsis*, *Solanum Lycopersicum*, *Zea may*, and *Oryza sativa*. It might be directly because that potassium deficiency reduced the photosynthesis efficiency absorbed light energy excess leading to ROS production. But not all plants exhibit oxidative stress as a result of potassium deprivation. Tobacco is an experimental model of plant science, and it is also a vital horticultural crop worldwide. Potassium is the essential quality element of tobacco (Sims, 1985). However, the mechanical research on tobacco responding to varied K^+^ concentrations is lacking.

LncRNAs are RNAs longer than 200 nucleotides and without potential coding ability. In almost all eukaryotic species and tissues, amounts of LncRNAs need to be found. LncRNAs have been generally classified into three types according to their genomic locations: long intergenic ncRNAs (lincRNAs) in the intergenic regions, intronic ncRNAs in the intronic regions, and natural antisense transcripts (NATs) from the antisense coding regions (Mercer et al., 2009). Their functions yet remain largely unknown. However, more and more studies have shown that some LncRNAs play a vital role in some biological progress (Chen et al., 2021). For instance, in *Arabidopsis*, several functional LncRNAs were identified and involved in cell phosphate homeostasis (Shin et al., 2006); in strawberry, one LncRNA was identified to play a role in fruit ripening (Tang et al., 2021). Functional LncRNAs were also detected in other plant species, for example, rice, maize, lemon, etc., (Chen et al., 2021). In the Solanaceae family, functional LncRNAs were identified only in tomato (*Solanum Lycopersicum*) and relevant with disease resistance, hormone signal pathway, and nitrogen deficiency (Cui et al., 2017; Wang et al., 2018; Jiang et al., 2020). There were 160 functional LncRNAs discovered in the model plant *Arabidopsis*, which, however, is the plant species with the most abundant functional LncRNA information. Meanwhile, few studies on LncRNA of potassium starvation in plants, especially in tobacco, were reported. The present research focused on the relationship between potassium deficiency and oxidative stress and potential regulatory crosstalk between coding RNAs and long non-coding RNAs in tobacco seedlings. The relevant physiological parameters and gene expression were monitored. The transcriptomic profile of coding gene RNA and the long non-coding RNA was addressed, and a collaborative gene expression regulation network was constructed.

## Materials and Methods

### Plant Materials and Low K Treatment

The tobacco cultivar “NC89” (*Nicotiana tabacum* L.) was used as an experimental subject. Seeds were germinated in trays filled with a mixture of peat and vermiculite (V/V, 1:1) held in an incubator under natural light at temperatures of 28/22°C for day and night. The 25-day seedling age of uniform size and vigor was transplanted into holes in a lid placed over the top of 10-L pots (15 holes per lid and one seedling per hole). One-quarter strength of Hoagland’s nutrient solution was provided for 14 days (Hoagland, 1950). Seedlings were subjected to two treatments: K^+^ deficiency (LK,0.01 mM) or normal nutrition (control, 2 mM). Potassium was supplied in the nutrient medium as K_2_SO_4_. The nutrient solution (pH 6.0) consisted of 1.25 mM Ca(NO_3_)^2^, 0.25 mM NaH_2_PO4, 0.5 mM MgSO_4_⋅7H_2_O, 20.0 μM Fe-EDTA, 9.1 μM MnCl_2_, 0.5 μM (NH_4_)_6_Mo_7_O_24_, 46 μM H_3_BO_3_, 0.8 μM ZnSO_4_, and 0.3 μM CuSO_4_. The nutrient solution was replaced daily. Each treatment was replicated 6-folds and arranged in a completely randomized design to avoid edge effects. In addition, all experiments included three independent biological replicates. Root and shoot samples were snap-frozen in liquid nitrogen and stored in a freezer at −80°C until the activity of active oxygen and the antioxidant enzyme was measured.

### Measurements of Malondialdehyde, Superoxide Anion, and H_2_O_2_

The MDA concentration in tobacco roots and shoots was measured as described by Wu et al. (2003) (Wu et al., 2003). Superoxide anion concentration in tobacco roots and shoots was measured as described by Wang et al. (2008) (Wang et al., 2008). H_2_O_2_ concentration was measured using Titanium Sulfate Assay (Ferguson et al., 1983).

### Measurement of Antioxidant Enzyme Activity

We analyzed the activities of superoxide dismutase (SOD) (EC 1.15.1.1), POD (EC 1.11.1.7), and APX (EC 1.11.1.11) using an assay kit (Shanghai Solarbio Bioscience & Technology Co., LTD., Shanghai, China) and made some adjustments to the method. SOD activity unit (U): In the xanthine oxidase coupling reaction system, when the inhibition percentage is 50%, the SOD enzyme activity in the reaction system is regarded as an enzyme activity unit and calculated using the fresh weight of the sample. POD activity unit (U): One unit (U) was defined as the amount of enzyme extract that caused an increase in absorbance of 0.01 per minute at A470. APX activity unit (U): Each mg of tissue oxidizes 1 μmol of ascorbic acid per minute as an enzyme activity unit. The ELISA kit (Randox Total Antioxidant Status, United Kingdom; Cat No: NX 2332) was utilized to quantify total antioxidant capacity (TAC) according to the manufacturer’s manual. The peroxidase substrate 2,2′-Azino-di-(3-ethylbenzothiazoline)-6-sulfonic acid, ABTS, was incubated with H2O2 and metmyoglobin peroxidase to obtain the radical form of ABTS. The ABTS cation had a relatively uniform blue-green color, which was read at the wavelength of 600 nm. Antioxidants in the samples suppressed the color production, proportional to their concentration. All the enzyme activities were expressed as U/g fresh weight.

### Transcriptome Sequencing and RNA-Seq Data Processing

Total RNA was extracted with the RNAprep Pure Plant Kit (TIANGEN, 432, China) and sequenced using Illumina HiSeq X Ten system with paired-end method by Annoroad Gene Technology (Beijing) Co. Ltd. For transcriptome analysis, the reference genome version and annotation files of Edwards et al. (2017) were employed in the study (Edwards et al., 2017). All RNA-seq datasets were first trimmed using Fastp v0.20.0 (Chen et al., 2018) with sequencing adapters, low-quality bases, and too short reads (<50 bp). Cleaned data were then aligned against the reference genome using STAR v2.7.9a with two-pass mode (Dobin and Gingeras, 2016). The mapped reads were then assembled by the reference annotation-based transcript (RABT) assembly algorithm. A combined GTF-formatted file with known transcript annotation was generated using StringTie v2.1.5 (Pertea et al., 2016). Finally, the expression value of transcripts was quantified as counts with the above-updated annotation using featureCounts v2.0.3 (Liao et al., 2014) and then normalized as FPKM (fragments per kilobase of transcript per million fragments mapped) value by custom Perl script. Only the transcripts with an FPKM > 1 in at least three samples were used for downstream differential expression analysis.

### Computational Prediction of LncRNAs

To identify potential LncRNAs, a strict computational strategy was performed as described by Lv et al., 2019 (Lv et al., 2019). First, all transcript sequences were extracted by Gffread v0.12.2 program (Geo and Mihaela, 2020). Second, we employed two tools, CPC2 v2.0 (Kang et al., 2017)and PLncPRO v1.2.2 (Urminder et al., 2017), to predict the coding potential of every transcript. Swissport and Pfam protein databases were selected for the PLncPRO program. CPC2 and PLncPRO were then performed with default parameters. Finally, non-coding transcripts larger than 200 bp, with an FPKM > one and joint from two tools, were considered as candidate LncRNAs.

### Differential Gene Expression Analysis

DESeq2 v1.32.0 (Love et al., 2014) used pairwise comparisons between conditional samples to identify differentially expressed genes (DEGs) with the combined transcript annotation. In this study, transcripts including coding and non-coding RNAs were considered as differentially expressed transcripts according to the following criteria: (I) Log_2_(fold-change) should be > 1; (II) the adjusted *p*-value from DESeq2 analyses had to be < 0.05.

### Co-expression Network Analysis

The above count matrix under conditions was employed to link the coding and non-coding RNAs to construct the weighted co-expression network by the WGCNA program v1.70 (Pei et al., 2017). The raw count expression matrix was normalized by the DESeq2 package (Love et al., 2014). Co-expression correlation between coding and non-coding RNAs was then calculated using Pearson’s correlation with R^2^ ≥ 0.85. The normalized expression data from coding and non-coding RNAs were extracted to construct an unsigned co-expression network using the WGCNA package with a soft threshold = 6. Module assignment of coding and non-coding RNAs was identified using a topological overlap matrix (TOM). Besides, the correlation between modules and treatments was also calculated, and transcripts from significantly correlated modules were extracted and visualized by Cytoscape v3.8.2 (Saito et al., 2012). Furthermore, the correlation value of module-trait and membership within modules are calculated by signedKME function of WGCNA package, and transcripts with module membership (MM) value larger than 0.8 were considered as hub genes. The hub gene is usually defined as the gene with the highest degree of connectivity in the key module, which may play an essential role in gene regulation and biological processes. A set of candidate hub lncRNAs in K^+^-responsive modules by computational prediction were provided for further molecular functional studies.

### Gene Ontology Enrichment Analysis

To infer the potential biological function of LncRNAs, coding transcripts in modules related to differences were then performed for gene ontology (GO) enrichment analysis using AgriGO v2.0 toolkit (Tian et al., 2017). Significantly, overrepresented GO terms were detected *via* Fisher’s exact test. Multi-test adjustment was made using Yekutieli (FDR under dependency) method with a cutoff of FDR < 0.05. GO enriched results were combined and visualized by clusterProfilerv4.0.4 (Yu et al., 2012).

### RNA Extraction and qRT-PCR Validation

Total RNA was extracted using the RNAprep Pure Plant Kit (Tiangen, China) according to the kit instructions, and cDNA was synthesized using PrimeScript RT Master Mix (Takara, Japan) according to the kit instructions. PCR reactions were performed using the SYBR^®^ Premix DimerEraser™ kit (Takara, Japan) on the LightCycler 96 Real-Time PCR System (Roche, United States). Each PCR reaction was done three times. The relative expression levels of transcripts were measured and normalized using the comparative Ct (ΔΔCt) method compared to a reference gene *NtEF1*α gene (Kajikawa et al., 2017). qPCR primers were designed using Beacon Designer 8 software (Horejsh et al., 2005) and found in the additional file: Supplementary Table 7.

## Results

### Oxidative Stress Physiological Parameters

**FIGURE 1:**
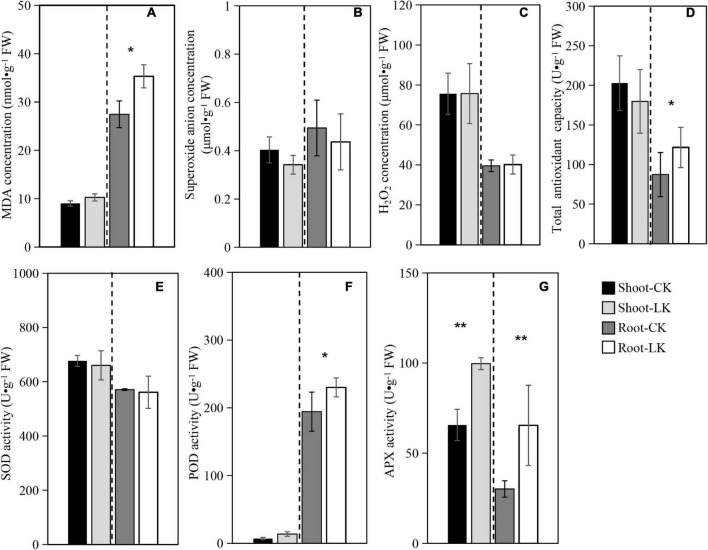
Oxidative stress physiological parameters. **(A)** Malondialdehyde (MDA); **(B)** superoxide anion (OFR); **(C)** hydrogen peroxide (H_2_O_2_); **(D)** superoxide dismutase (SOD); **(E)** peroxidase (POD); **(F)** ascorbate-peroxidase (APX); **(G)** total antioxidant capacity (TAC). Shoot-CK: shoot of NC89 in sufficient K^+^ treatment; Root-CK: the root of NC89 in sufficient K^+^ treatment; Shoot-LK: shoot of NC89 in low K^+^ treatment; Root-LK: the root of NC89 in low K^+^ treatment.

The MDA was assayed in shoots and roots of the tobacco seedlings subjected to low K^+^ treatment with the sufficient K^+^ concentration as control. As shown in Figure 1A, in comparison with controls, K^+^ deficiency significantly increased MDA concentration in tobacco roots. However, there was no significant difference in shoots. As shown in Figures 1B,E, in comparison with controls, K^+^ deficiency decreased superoxide anion concentration and SOD activity in shoots and roots. However, we detected no significant differences between the two treatments, and the H_2_O_2_ concentration was also not obviously different between the different treatments (Figure 1C).

In comparison with controls, K^+^ deficiency significantly increased POD activity in tobacco roots. However, there was no significant difference in shoots (Figure 1F). APX activity was also assayed, as shown in Figure 1G. The low K^+^ treatment promoted APX activity in roots and shoots of the tobacco seedling to significantly higher levels.

Finally, TAC was assayed, as shown in Figure 1D. The results showed that the low K^+^ treatment significantly promoted TAC in tobacco roots from 87.37 (control in average) to 121.66 Ug^–1^ FW (low K^+^). On the other hand, no significant difference was detected between low and sufficient K + shoots.

### Reference-Guided Assembly and Identification of Long Non-coding RNAs

A total of 12 datasets of transcriptome were employed, with two potassium concentration treatments (sufficient and low) and each treatment with three replications and the roots and shoots were separated. The raw data were assembled with reference tobacco genome. A total of 91,964 transcripts were generated. Among them, 35,372 were exactly matched the reported coding genes.

**FIGURE 2:**
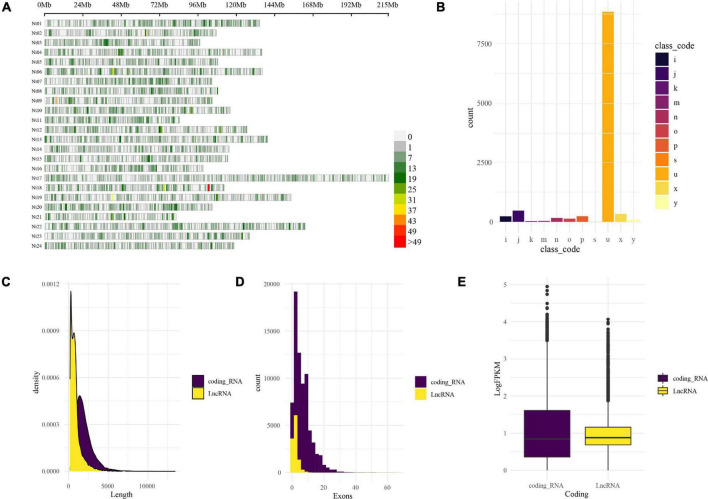
Genome-wide identification and characterization of LncRNA in tobacco under low K. **(A)** LncRNA distribution on the tobacco reference genome. The LncRNA distribution and density are demonstrated by the chromosomes’ physical positions and coloration; the window size is 1-Mb base pairs. **(B)** Physical position compared with reference annotation. The transcripts were classified by GffCompare software into different groups in different colors and marked with characters and symbols. For example, “u,” intergenic; “i,” fully contained within intron; “j,” multi-exon with at least one junction match; “o,” other same strand overlap with reference exons; “p,” possible polymerase run-on; “x,” exonic overlap on the opposite strand; “s,” intron match on the opposite strand; “k,” containment of reference; “m,” retained intron, all introns matched; “n,” retained intron, not all introns matched; “y,” contains a reference within its intron; **(C)** length distribution of coding RNAs and LncRNAs. **(D)** Exons distribution of coding RNAs and LncRNAs. **(E)** The expression abundance (logFPKM) of coding RNAs and LncRNAs.

LncRNAs were identified by CPC2 and PLncPRO, respectively. A total of 11,472 transcripts were identified as LncRNAs. The distribution of LncRNAs on the tobacco chromosomes is demonstrated in Figure 2A. The resource of these LncRNAs could be considered into 11 types, as shown in Figure 2B. The top type was the “u” type, also named lincRNA (long intergenic non-coding RNA), which contained 8,853 transcripts from the intergenic region. The following one was “=” type, including 888 transcripts, which represents annotated transcripts previously. The rest types such as “i,” “j,” “k,” “m,” “n,” “o,” “p,” “s,” “x,” and “y” are described in Figure 2 legend. Compared with the coding genes, the length of LncRNAs was shorter than the coding genes, which is demonstrated in Figure 2C. Besides, the exon numbers in most LncRNAs were one or two (Figure 2D), and LncRNAs have relatively lower expression abundance than coding RNAs (Figure 2E). The characteristic of LncRNAs was unique and distinct from coding genes.

### Differentially Expressed Coding RNAs and LncRNAs

**FIGURE 3:**
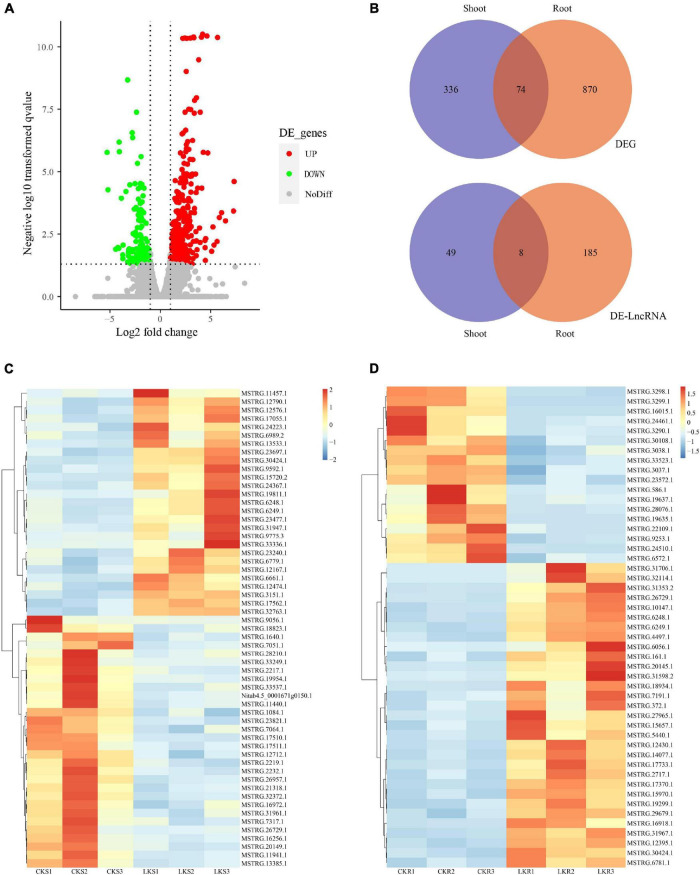
The expression profile of coding RNAs and LncRNAs under low K^+^. **(A)** The volcano plot of differentially expressed coding RNAs and LncRNAs. **(B)** Venn plot of coding RNAs and LncRNAs, and overlap between shoot and root; **(C)** the heatmap of shoot-specific DE-LncRNAs under low K treatments; **(D)** the heatmap of root-specific DE-LncRNAs under low K treatments.

The DEGs and DE-LncRNAs were identified further according to the expression level calculated with the FPKM value. The result showed that 944 DEGs and 193 DE-LncRNAs are placed in the root and 410 DEGs and 57 DE-LncRNAs in the shoots between sufficient and low potassium treatments. The distribution of DEGs and DE-LncRNAs is also shown in Figure 3A. A number of 74 DEGs were overlapping between the shoot and root when eight LncRNAs were overlapping (Figure 3B). The represented DE-LncRNAs in shoot and root were further shown as a heatmap in Figures 3C,D. Overall, the amount of K^+^-responsive DEGs and DE-LncRNAs was identified, which indicated that coding and long non-coding RNAs might coordinately play a vital role in low K^+^ response.

### Co-expression Network Construction With Coding and Long Non-coding RNAs

**FIGURE 4:**
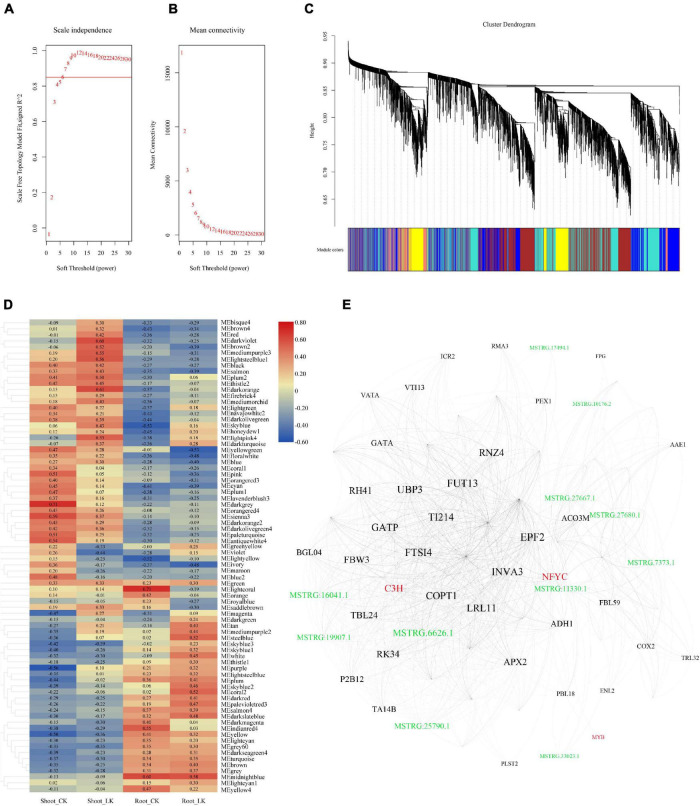
The co-expression network was constructed combined with expressed LncRNAs and coding genes. **(A)** Analysis of the scale-free fit index for soft-thresholding powers from 1 to 30; **(B)** analysis of the mean connectivity for soft-thresholding powers from 1 to 30; **(C)** the clustering dendrogram with dissimilarity based on the topological overlap, together with assigned module colors; **(D)** the heatmap of correlation between co-expression modules and the treatments; **(E)** network illustration of co-expression modules associated with low K treatment. The spots represent the node genes, which have high intramodular connectivities. Transcription factors are marked as red, and the spots in green are LncRNAs.

To further illustrate the potential crosstalk between coding RNA and LncRNA in the tolerance of low potassium stress, DEGs and DE-LncRNAs described above were linked according to their expression profiles. WGCNA generated the co-expression networks, and the parameters about scale-free fit index and mean connectivity of soft-thresholding power were calculated from 1 to 30 and demonstrated in Figures 4A,B. A total of six were then defined as the power value for network construction. A total 70 modules were finally generated and clustering dendrogram based on topological overlap, along with module colors allocated, was shown in Figure 4C. According to the size of which the genes and LncRNAs included, the most prominent part was the gray containing a total of 6,030 genes and 1,345 LncRNAs, which were unassigned into any module; the following module is “turquoise,” which consisted of 4,119 genes and 1,371 LncRNAs; the third one is “blue” module including 3,707 genes and 875 LncRNAs. The correlation between the modules and the low potassium treatment is demonstrated in Figure 4D. When the threshold of the correlation value with the low potassium treatment was set as ≤ −0.5 or ≥ 0.5, there were only nine K^+^-responsive modules left including 616 coding RNAs and 146 LncRNAs: “steelblue,” “lightsteelblue1,” “lightpink4,” “darkorange,” “darkviolet,” “mediumpurple3,” “yellowgreen,” “coral2,” and “brown2” (Supplementary Table 5). By further MM analysis, 158 transcripts, including 138 coding RNAs and 20 hub LncRNAs, were considered hub genes with higher connectivity and may play an important role in the expression regulatory network (Supplementary Table 5). Eigengenes from these modules could be combined and shown in Figure 4E. There were 75 nodes in this network, including three transcript factor genes, such as the MYB family, C3H family, NFYC family, and 11 LncRNAs. These results suggested that LncRNAs may interact with transcript factors and play a hub role in low K^+^ response.

### Gene Ontology Enrichment of K^+^-Responsive Modules

**FIGURE 5:**
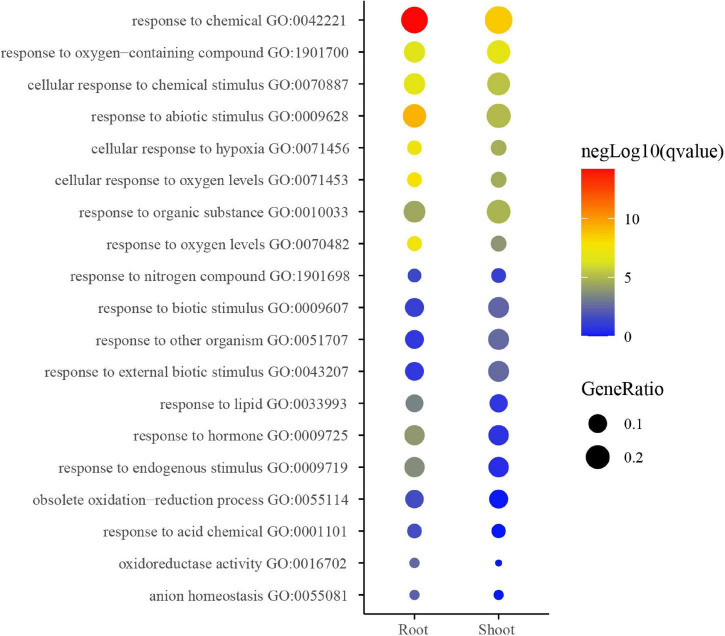
GO analysis of low K-responsive modules in root and shoot. The heatmap scale reflects the significant level of enrichment of GO terms; the red showed the most significant enrichment in statistics. Size of spot represents transcript numbers of terms.

Gene ontology enrichment analysis of coding genes within the above-identified K^+^-responsive modules was then performed for inferring the potential biological function of low K^+^-responsive LncRNAs. The enrichment result shows the main terms in Figure 5. A part of the terms was significantly enriched in both shoot and root, such as responding to stimulations (GO:00770887, GO:0009628, GO:0009607, GO:0009719). Among them, there were three pathways relevant to oxygen (GO:0071456, GO:0071453, and GO:0070482). On the other hand, the pathways of the obsolete oxidation-reduction process (GO:0055114), oxidoreductase activity (GO:0016702), and response to hormone (GO:0009725) were more significant in the root. Besides, the response to nitrogen compound (GO:1901698) was also enriched.

### qRT-PCR Validation of the Differentially Expressed LncRNAs

**FIGURE 6:**
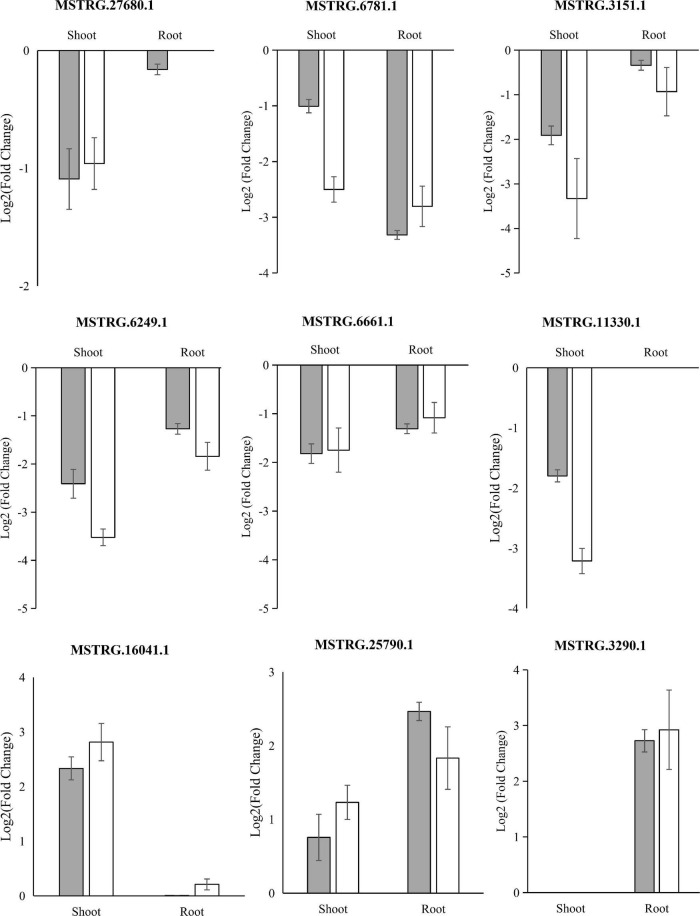
qRT-PCR validation of differentially expressed LncRNAs. Mean ± SE (standard error) of three independent biological replicates was shown and compared with a fold-change value between RNA-seq groups.

To validate the reliability of low K^+^-responsive LncRNAs, we performed an independent treatment experiment, and their RNA was extracted. We then subjected the samples to quantitative real-time PCR (qRT-PCR) to compare expression changes between replicated control and low K-treated. We randomly selected eight long, non-coding transcripts and five low K^+^-responsive hub LncRNAs: four failed due to primer specificity; three LncRNAs, including two hub LncRNAs, were significantly upregulated in root under low K^+^ treatment based on qRT-PCR; six LncRNAs, including two hub LncRNAs, were significantly downregulated in shoot and root under low K^+^ treatment (Figure 6). Quantitative results showed a high degree of consistency with the expected RNA-seq (Figure 6 and Supplementary Table 7).

## Discussion

Potassium is crucial for plant growth (Wang et al., 2021). In this study, we had carried out a series of tobacco hydro-cultivation experiments with sufficient concentration and low K^+^ treatment in the liquid medium. It has been well studied and documented that potassium deficiency could induce oxidative stress. The reactive oxidative species (ROS) resource is primarily the photosynthesis system (Demidchik, 2015). The lack of potassium would lower the photosynthetic efficiency, and the residue energy leaks as ROS, which is harmful to plant cells and tissue (Tränkner et al., 2018). Lipid peroxidation is quite a common consequence of oxidative stress in the plant. A series of oxidation reactions of the cell membrane system constituted of a phospholipid, and the terminal product is MDA. Lipid peroxidation could damage cellular organs such as chloroplast and mitochondria, as well as subcellular functional structures, for example, the endoplasm reticulum (Anderson, 1995; Demidchik, 2015). Previously, many studies on potassium starvation in tobacco were reported (Lu et al., 2015; Hu et al., 2019). The result showed that low K^+^ in fertilization would inhibit lateral root development (Song et al., 2015), lower starch accumulation in leaves (Day, 1940), and other symptoms. Transcriptomic research on tobacco leaves with potassium starvation treatment showed that the K^+^ deficiency promotes the antioxidant activity and structural molecule activity significantly (Lu et al., 2015; Li et al., 2016).

However, there is no LncRNA study report on potassium deficiency in tobacco. In the study, we provided massive information for candidate LncRNAs response to low K^+^ stress in tobacco. Simultaneously, this study also proposed the potential networks of LncRNA and coding genes, which provided clues for further studies on LncRNA and coding genes interaction. As a result, nine K^+^-responsive modules, including 616 coding RNAs and 146 LncRNAs, were identified, and 20 hub LncRNAs were further predicted by MM ranking. The transcriptome showed that the potassium transporter at gene expression level promoted by potassium starvation, and the gene is in module “black,” and in this module, there were five LncRNAs. The physiological analysis on the plant material indicated that although there was no significant ROS level elevation in the shoot or root part of plantlet in low K^+^ cultivation, an increase in MDA was detected. Meanwhile, the activity of APX in low K^+^ material was also increased both in the shoot and root. These results implied that potassium’s starvation might induce oxidative stress, but the antioxidative enzyme system was triggered, and the ROS was scavenged off.

Interestingly, we also assayed antioxidative enzyme activities of SOD, but there was no increase due to the low K^+^ treatment. This phenomenon thus implied that the potassium-induced ROS explosion might be scavenged by APX and POD specifically. The transcriptomic data also agreed with the physical result. The expression profile also showed that only APX and POD coding genes’ expression abundance increased, but not other antioxidative enzymes. Besides, the result is also portrayed in the root. The coding genes of the antioxidative enzyme were expressed only in PODs. A total of 14 POD coding genes in the root had significantly higher expression abundance after potassium starvation; meanwhile, the expression abundance of one *POD* and one *APX* gene elevated in the shoot. Hence, the dataset of LncRNAs provided by the present research should also include those regulating POD and APX antioxidative enzyme systems. For instance, in the result, one network indicated in Figure 4E was generated from the modules with a correlation value fulfilling the threshold. In this network, there is one *APX2* coding gene (MSTRG.1257.1), of which low K^+^ promoted the expression in roots, included as a node. The module containing this *APX2* coding gene was the “lightpink4” module (Supplementary Table 5), and there were four LncRNAs.

The GO analysis also showed the significant enrichment in the pathways of obsolete oxidation-reduction process, oxidoreductase activity (Figure 5), which also agreed with the physiological results. On the other hand, many pathways on the top were to some degree related to stress, chemical, and it also implied that potassium starvation could negatively affect the seedlings. Moreover, oxygen and hypoxia pathways were enriched, as shown in Figure 5. This result could be caused by the hydro-cultivation method employed in this research. Tobacco is a soil-growing plant, and laboratory hydro-cultivation could easily cause a lack of hypoxia and induce the expression of relevant genes. It could influence the result to some degree.

Overall, the present research carried out a potassium starvation experiment in a tobacco plantlet. With transcriptomic sequencing, the dataset of coding gene and LncRNA was obtained. Further analysis generated the co-expression network modules. These modules provide clues that functional LncRNAs are involved in regulating K^+^ absorbing and transport and APX-mediated ROS scavenging.

## Data Availability Statement

The datasets presented in this study can be found in online repositories. The names of the repository/repositories and accession number(s) can be found below: The RNA-Seq data were deposited in the NCBI SRA database (Accession number: PRJNA744321); Bioinformatic scripts in the study are at https://github.com/lyd0527/LncRNA_KUE.

## Author Contributions

WS and YL conceived and designed the experiments and revised the manuscript. XC and LM performed the experiments and wrote the manuscript. BH, WQ, LJ, NX, and FH participated in data collection and analysis. All authors have read and approved the final manuscript.

## Conflict of Interest

The authors declare that the research was conducted in the absence of any commercial or financial relationships that could be construed as a potential conflict of interest.

## Publisher’s Note

All claims expressed in this article are solely those of the authors and do not necessarily represent those of their affiliated organizations, or those of the publisher, the editors and the reviewers. Any product that may be evaluated in this article, or claim that may be made by its manufacturer, is not guaranteed or endorsed by the publisher.

## Supplementary Material

Supplementary Table 1 Reference-guided assembly annotation based on RNA-seq from shoot and root under low K treatments.

Supplementary Table 2 The summary of potential LncRNAs.

Supplementary Table 3 The differentially expressed LncRNAs.

Supplementary Table 4 Modules of co-expression network and their correlation with low K treatments.

Supplementary Table 5 Candidate hub LncRNAs in nine K^+^-responsive modules.

Supplementary Table 6 GO enrichment result associated with tissues x treatments.

Supplementary Table 7 qRT-PCR primers.
